# MAFB is dispensable for the fetal testis morphogenesis and the maintenance of spermatogenesis in adult mice

**DOI:** 10.1371/journal.pone.0190800

**Published:** 2018-01-11

**Authors:** Hossam H. Shawki, Hisashi Oishi, Toshiaki Usui, Yu Kitadate, Walaa A. Basha, Ahmed M. Abdellatif, Kazunori Hasegawa, Risa Okada, Keiji Mochida, Hany A. El-Shemy, Masafumi Muratani, Atsuo Ogura, Shosei Yoshida, Satoru Takahashi

**Affiliations:** 1 Department of Anatomy and Embryology, Graduate School of Comprehensive Human Science, University of Tsukuba, Tsukuba, Japan; 2 Department of Animal Genetic Resources, National Gene Bank, Giza, Egypt; 3 Department of Comparative and Experimental Medicine, Graduate School of Medical Sciences, Nagoya City University, Nagoya, Japan; 4 Division of Germ Cell Biology, National Institute for Basic Biology, Okazaki, Japan; 5 Department of Medicine, Stanford University School of Medicine, Stanford, California, United States of America; 6 BioResource Center, RIKEN, Tsukuba, Japan; 7 Cairo University Research Park, Cairo University, Giza, Egypt; 8 Department of Genome Biology, Faculty of Medicine, University of Tsukuba, Tsukuba, Japan; Universite Clermont Auvergne, FRANCE

## Abstract

The transcription factor MAFB is an important regulator of the development and differentiation of various organs and tissues. Previous studies have shown that MAFB is expressed in embryonic and adult mouse testes and is expected to act as the downstream target of retinoic acid (RA) to initiate spermatogenesis. However, its exact localization and function remain unclear. Here, we localized MAFB expression in embryonic and adult testes and analyzed its gene function using *Mafb*-deficient mice. We found that MAFB and c-MAF are the only large MAF transcription factors expressed in testes, while MAFA and NRL are not. MAFB was localized in Leydig and Sertoli cells at embryonic day (E) 18.5 but in Leydig cells, Sertoli cells, and pachytene spermatocytes in adults. *Mafb*-deficient testes at E18.5 showed fully formed seminiferous tubules with no abnormal structure or differences in testicular somatic cell numbers compared with those of control wild-type mice. Additionally, the expression levels of genes related to development and function of testicular cells were unchanged between genotypes. In adults, the expression of MAFB in Sertoli cells was shown to be stage specific and induced by RA. By generating *Mafb*^fl/fl^ CAG-CreER^™^ (*Mafb*-cKO) mice, in which Cre recombinase was activated upon tamoxifen treatment, we found that the neonatal cKO mice died shortly upon *Mafb* deletion, but adult cKO mice were alive upon deletion. Adult cKO mice were fertile, and spermatogenesis maintenance was normal, as indicated by histological analysis, hormone levels, and germ cell stage-specific markers. Moreover, there were no differences in the proportion of seminiferous stages between cKO mice and controls. However, RNA-Seq analysis of cKO Sertoli cells revealed that the down-regulated genes were related to immune function and phagocytosis activity but not spermatogenesis. In conclusion, we found that MAFB is dispensable for fetal testis morphogenesis and spermatogenesis maintenance in adult mice, despite the significant gene expression in different cell types, but MAFB might be critical for phagocytosis activity of Sertoli cells.

## Introduction

The testes are divided into several tubules known as seminiferous tubules, which are the houses of sperm production. Each tubule comprises multiple germinal cell types and only one somatic cell type, Sertoli cells, which support sperm development. Leydig cells, another type of somatic cell, are located outside the tubules and produce androgens required for the maturation of sexual organs and sexual characteristics as well as sperm development. The testes produce sperm through a process known as spermatogenesis. Spermatogenesis is a complex process of cellular transformation that depends on numerous factors for successful production of haploid sperm from diploid spermatogonial stem cells [1].

Spermatogenesis comprises three main phases (mitosis, meiosis, and post-meiosis). Spermatogonia are diploid and divide by mitosis into several other types of spermatogonia. Spermatogonia are present as undifferentiated type A spermatogonia (A single, A paired, A aligned), which all retain stem cell properties; differentiated type A spermatogonia (A1, A2, A3, A4); intermediate spermatogonia; and type B spermatogonia. Type B spermatogonia are then divided by mitosis to form preleptotene, leptotene and zygotene spermatocytes, which subsequently undergo meiosis I to form secondary spermatocytes and meiosis II to form haploid round spermatids. Spermiogenesis is the post-meiosis process that transforms spherical, haploid spermatids into elongated spermatid and mature sperm that are released into the lumen of the seminiferous tubules.

MAF family of proteins is a subgroup of basic region-leucine zipper (bZIP) transcription factors that recognize a long palindromic DNA sequence [TGCTGAC(G)TCAGCA] known as MAF recognition element (MARE) [2]. The MAF family is subdivided into two groups; large MAFs and small MAFs. Large MAFs contain an acidic domain that promotes transcriptional activation. In contrast, small MAFs lack the acidic domain and act as transcriptional repressors unless they form heterodimers with other proteins that harbor transcriptional activation domains [3–6]. In mice, large MAFs include MAFA, MAFB, c-MAF and Neural Retina Leucine (NRL), while small MAFs include MAFF, MAFG, and MAFK. In *Drosophila melanogaster*, there is only one large MAF transcription factor called *Traffic Jam* (*TJ*) and one small MAF called MAF-S. Several studies have shown that each protein plays a key role in cellular differentiation and regulation of tissue-specific gene expression [7, 8].

Inactivation of *TJ*, the large MAF factor in *Drosophila*, revealed that it plays an essential role in gonad morphogenesis and that its expression in somatic gonadal cells in direct contact with germline cells is required for female and male fertility [9]. In *TJ* mutant gonads, somatic cells fail to intermingle and properly envelop germline cells, causing an early block in germ cell differentiation. *TJ* encodes an orthologue of the typical bZIP transcription factors MAFB and c-MAF in vertebrates.

In particular, the large MAF transcription factor in vertebrates, MAFB, is first expressed in mouse embryonic gonads along the gonad-mesonephros border in both sexes as early as embryonic day (E) 11.5. Between E12.0 and E14.5, MAFB expression expands in the interstitial compartment and then becomes restricted to Leydig cells in XY gonads, but the expression pattern does not change significantly in XX gonads [10]. On the other hand, MAFB in post-natal mouse testes has been detected in Sertoli cells within the seminiferous tubules [11] and in testicular macrophages outside the tubules [12].

The active metabolite of vitamin A, retinoic acid (RA), is essential for the initial differentiation and meiotic entry of spermatogonia. Vitamin A-deficient (VAD) mice result in blockage of A to A1 spermatogonia transition, and only undifferentiated type A spermatogonia and Sertoli cells remain within the seminiferous tubules in the testes [13]. This indicates that removing RA inhibits the ability of undifferentiated spermatogonia to differentiate in adult mouse testes. Treating VAD mice with retinol or RA results in the complete recovery of spermatogenesis [13]. Two models have been suggested for how RA drives male germ cell development [11, 14–19]. First, RA that generated by Sertoli cells in the neonatal testes acts in an autocrine manner to induce the first wave of A1 spermatogonia differentiation. Second, after the first wave, the transition of A to A1 spermatogonia appears to be generated by the activity of RA in either pachytene spermatocytes or preleptotene spermatocytes. However, the target genes that are regulated by RA and that control spermatogonia differentiation remain to be discovered.

A previous report revealed that MAFB is one of the RA target genes that induces spermatogonia differentiation [11]. The authors specifically knocked out RA-synthesizing enzymes (RALDH1 to RALDH3 encoded by *Aldh1a1* to *Aldh1a3* genes) in Sertoli cells and found a blockage of spermatogonial differentiation, similar to VAD mice. After treatment with a RARA agonist, differentiated spermatogonia were detected with highly increased MAFB expression in Sertoli cells [11]. In addition, they detected a robust RAR-binding site at the end of the *Mafb* coding region, indicating that this gene is a direct target of RA.

However, the complete role of MAFB in spermatogonia differentiation remains unclear because *Mafb* knockout mice die by birth, and conditional alleles are not generated by this time. In the current study, we generated conditional alleles of *Mafb* and investigated the role of *Mafb* in male gonads.

## Materials and methods

### Animals

All mice used in this study were of the C57BL/6 background. Animal experiments were approved by the Animal Experiment Committee of the University of Tsukuba (Permit Number: 14–049) and performed in accordance with the Guide for the Care and Use of Laboratory Animals of the National Institutes of Health. All the mice received humane care, were maintained in specific pathogen-free conditions and were euthanized with carbon dioxide gas in the Laboratory Animal Resource Center at the University of Tsukuba to minimize suffering. Animals were checked daily and the specific criteria for animal euthanasia when they displayed early markers associated with death or specific signs of severe suffering or distress, including slow or no mobility or an absence of heartbeat or respiratory movement.

Wild-type (WT) mice were purchased from the Institute for Animal Reproduction (Kasumigaura-shi, Japan). *Mafb*/GFP knock-in null mutant (*Mafb*^−/−^) *embryos* were generated and genotyped as previously described [20]. *Mafb* conditional knockout (*Mafb*-cKO) mice were generated by homologous recombination in embryonic stem (ES) cells. Briefly, the mouse *Mafb* locus was cloned from a BAC clone, and the targeting construct was linearized and transfected into ES cells by electroporation. Recombinant ES cell clones expressing the neomycin gene were selected and injected into C57BL/6 mouse-derived blastocysts using standard procedures. The neomycin selection cassette was flanked by FRT recombination sites and excised *in vivo* by crossing with the general FLP deleter strain (Jackson Laboratories). Floxed heterozygous *Mafb*^fl/+^ and heterozygous CAG-CreER^™^ mice (Jackson Laboratories) were crossed to generate double heterozygous *Mafb*^fl/+^;CAG-CreER^™^ mice, which were bred with homozygous *Mafb*^fl/fl^ mice to produce conditional *Mafb* homozygous (*Mafb*^fl/fl^;CAG-CreER^™^) mice, *Mafb* heterozygous (*Mafb*^fl/+^;CAG-CreER^™^), homozygous *Mafb*^fl/fl^, and heterozygous *Mafb*^fl/+^ mice. Control mice lacked the Cre transgene (*Mafb*^fl/fl^). To induce gene deletion, mice were injected with tamoxifen. The primer sequences used for genotyping of *Mafb*^−/−^, *Mafb*^*loxP*^, and *Mafb* excision (*Mafb*^*Δ*^) are listed in Table 1.

**Table 1:** Complete list of primers used in this study.

Primer	Forward (5`- 3`)	Reverse (5`- 3`)
**RT-PCR**		
*Mafa*	CACTGGCCATCGAGTACGTCA	CTTCACCTCGAACTTCATCAGGTC
*Mafb*	TGAATTTGCTGGCACTGCTG	AAGCACCATGCGGTTCATACA
*c-Maf*	CTGCCGCTTCAAGAGGGTGCAGC	TCGCGTGTCACACTCACATG
*Nrl*	AACTT CTGA GCATC GTGGCA	TGAAGAGTCGTGACCTGCAAA
*Hprt*	TTGTTGTTGGATATGCCCTTGACTA	AGGCAGATGGCCACAGGACTA
**Genotyping**		
*Cre*	GAACCTGATGGACATGTTCAGG	AGTGCGTTCGAACGCTAGAGCCTGT
*GFP*	AAACGGCCACAAGTTCAG	GAAGTTCACCTTGATGCC
*Mafb*^*−/−*^	TGAGCATGGGGCAAGAGCTG	CCATCCAGTACAGGTCCTCG
*Sry*	TTGTCTAGAGAGCATGGAGGGCCATGTCAA	CCACTCCTCTGTGACACTTTAGCCCTCCGA
*Mafb* ^*loxP*^	AGGGTATGACTGTGTGTGCT	CAAGCCAGAATGCAAAAGCG
*Mafb*^*Δ*^	GCTTCTCTACCCTGCCCC	GGCCAAGCCTTTGTCTGG
**qRT-PCR**		
*Oct4*	TATTGAGTATTCCCAACGAGAAGAG	CTCAGGAAAAGGGACTGAGTAGAGT
*Nanos3*	CGGCCTGACAAGGCAAAGAC	CACCATGGTCCTCCCCACTC
*Kit*	AGCGTCTTCCGGCACAACGG	GCCAATGAGCAGCGGCGTGA
*Sohlh1*	GGGCCAATGAGGATTACAGA	CACAGGAGCTGTGCAGAGAG
*Stra8*	GGAGAAAAAGGCCAGACTCC	CCACGTCAAAAGCATCTTCA
*Prm2*	TGCAGGAAATGTAGGAGGCACCAT	AGGGCTCAGACATCGACATGGAAT
*Amh*	CTATTTGGTGCTAACCGTGGACTT	AAGGCTTGCAGCTGATCGAT
*Sox9*	ACAGATCTCCTACAGCCCCTTCAA	GCCGGAGTTCTGATGGTCAGCGTA
*Wt1*	GAGAGCCAGCCTACCATCC	CAACTGTCAGTAGGGGTGTGG
*Cyp17a1*	TGACCAGTATGTAGGCTTCAGTCG	TCCTTCGGGATGGCAAACTCTC
*StAR*	CGGGTGGATGGGTCAAGTTC	CCAAGCGAAACACCTTGCC
*Insl3*	TGCAGTGGCTAGAGCAGAGA	GTGCAGCCAGTAAGACAGCA
*Hsd3b1*	TGGACAAAGTATTCCGACCAGA	GGCACACTTGCTTGAACACAG
*Cyp11a1*	ACATGGCCAAGATGGTACAGTTG	ACGAAGCACCAGGTCATTCAC
*Gng11*	CTTCACATCGAGGATCTGC	TTTAGATACCTGTTGTCTCTGC
*Nck2*	CGTGTCTCTCAAAGCGTCAG	CCAATGGCTGCTTTCACTGT
*Prr3*	CGCTATGCCGAAACGAAAGA	GTGGTCCTTAACTGTTGGCC
*Tlr1*	CACCCCTACAGAAACGTCCT	TCCTTGGGCACTCTGGTAAG
*Nlrp3*	ATGCTGCTTCGACATCTCCT	AACCAATGCGAGATCCTGAC
*Hprt*	TTGTTGTTGGATATGCCCTTGACTA	AGGCAGATGGCCACAGGACTA

### Cre driver mice efficiency

R26GRR reporter mice, as previously described [21, 22], were mated with the CAG-CreER^™^ driver mouse strain obtained from Jackson laboratories. The progeny were genotyped using the Cre and GFP primers listed in Table 1. Frozen sections from the mice carrying double heterozygous mice carrying GRR and Cre recombinase were then analyzed with or without tamoxifen injection.

### Tamoxifen administration

Tamoxifen (Sigma; T5648) was dissolved in corn oil. The tamoxifen-oil mixture was stored at −20°C until used. Neonatal mice received 50 μg tamoxifen/day intragastrically from P1 to P3 [23]. Starting 6 weeks after birth, mice received 75 mg tamoxifen/kg body weight intraperitoneally once daily for 5 consecutive days [24]. All littermates were injected with the same dose to generate the necessary controls.

### Reverse transcription PCR (RT-PCR)

Total RNA was extracted from various tissues with a Nucleospin RNA II kit (TaKaRa 740955), and cDNA was synthesized with a QuantiTect Reverse Transcription kit (QIAGEN; 205313), each according to the manufacturer's instructions. PCR was performed for *Mafa*, *Mafb*, *c-Maf*, and *Nrl* using TaKaRa Ex Taq (RR001). *Hprt* was used as an internal control for cDNA quality and PCR efficiency. The sequences of the primers used for RT-PCR are shown in Table 1.

### Quantitative RT-PCR (qRT-PCR)

Total RNA was extracted from the testes with a Nucleospin RNA II kit (TaKaRa; 740955), and cDNA was synthesized with a QuantiTect Reverse Transcription kit (QIAGEN 205313), each according to the manufacturer's instructions. Quantitative PCR reactions were run on a Thermal Cycler Dice Real-Time System Single (Takara) with SYBR Green PCR master mix (Takara; RR820). All qPCR analyses were performed in duplicate. Amplification products were quantified by the standard curve method. The mRNA levels of each gene were normalized to that of *Hprt*. The primers used for qPCR are listed in Table 1.

### Tissue collection and histological analysis

Tissues were dissected from mice immediately after euthanasia, fixed in 4% (mass/vol) paraformaldehyde for up to 24 h (5 h in case of embryos and neonatal mice), stored in 70% (vol/vol) ethanol, and embedded in paraffin. Sections of 5-μm thickness were prepared and mounted on glass slides. After deparaffinization, slides were used either for immunohistochemical analyses or stained with periodic acid–Schiff (PAS) and haematoxylin/eosin (HE).

### Immunohistochemical analysis

Sections were deparaffinized, boiled for antigen retrieval in 20 mM citrate buffer (pH 6.0) for 10 min, and blocked in the appropriate serum for one hour. Sections were then incubated with a 1:400 dilution of the following antibodies overnight at 4°C: rabbit polyclonal anti-MAFB (BETHYL; IHC-00351), goat polyclonal anti-GATA4 (Santa Cruz Biotechnology; sc-1237), guinea pig polyclonal anti-vimentin (Progen Biotechnik; GP53), rabbit polyclonal anti-SOX9 (Santa Cruz Biotechnology; sc-20095), rabbit polyclonal anti-STAR (CST; 8449), goat polyclonal anti-GFRA1 (R&D; AF560), goat polyclonal anti-E-cadherin (R&D; AF748), rabbit polyclonal anti-PLZF (BETHYL; IHC-22839), mouse polyclonal anti-PLZF (Calbiochem; OP128), goat polyclonal anti-KIT (R&D; AF1356), and/or mouse polyclonal anti-SCP3 (Abcam; ab97672), followed by incubation with a 1:1000 dilution of the appropriate Alexa Fluor-conjugated secondary antibody (Life Technologies, Gaithersburg, MD, USA). In the case of double staining with PNA-lectin, a FITC-conjugated peanut agglutinin (Sigma; L7381) was incubated together with the appropriate secondary antibody. For double staining with two primary antibodies from the same host species (rabbit), Zenon Alexa Fluor 488 and 594 rabbit IgG1 labelling kits were used (Thermo Fisher Scientific). Sections were counterstained with DAPI.

### Immunocytochemical analysis

Isolated Sertoli cells were plated onto a silanized glass slide using a Shandon Cytospin^™^ 3 centrifuge. Cells were then fixed with 4% paraformaldehyde in PBS for 15 min at room temperature. Cells were washed with PBS and blocked with 3% skim milk plus 0.1% Triton X-100 in PBS. Cells were then incubated with a 1:500 dilution of guinea pig polyclonal anti-vimentin antibody (Progen Biotechnik; GP53) overnight at 4°C. After washing, cells were incubated with a 1:1000 dilution of Alexa Fluor 594-conjugated secondary antibody (Life Technologies, Gaithersburg, MD, USA) for an hour at room temperature. Cells were counterstained with DAPI.

### Counting of Leydig and Sertoli cells

Leydig and Sertoli cells were identified in 18.5-dpc mouse testes. Testes from WT and *Mafb* KO embryos (*n* = 3) were sectioned, and 4 sections for each gonad were randomly selected and stained with an antibody against GATA4 or STAR. The number of STAR-positive cells outside seminiferous tubules (Leydig cells) and GATA4-positive cells inside the tubules (Sertoli cells) were counted, and the whole gonadal areas were measured.

### Isolation of stage-specific seminiferous tubules

The specific stages of seminiferous tubules were isolated for qPCR analysis. Briefly, mature WT male mice were sacrificed, and the testes were dissected and decapsulated in a Petri dish containing PBS. The tubules were viewed on a trans-illuminating dissection microscope. The light absorption pattern was used to identify the different stages as previously described [25].

### Primary Sertoli cells culture

Primary Sertoli cells were isolated and cultured as previously described [26, 27] with slight modifications. Briefly, three-week-old testes without tunica albuginea were sequentially treated with 0.5 mg/ml collagenase (Wako, 034–22363), 1 mg/ml hyaluronidase (Sigma, H3506) plus 1 mg/ml collagenase, and 1 mg/ml hyaluronidase in Dulbecco's modified Eagle's medium (DMEM) containing DNase I. Small pieces of seminiferous tubules were removed via filtering through a 100μm-pore-size filter. The purity of the isolated cells was confirmed by immunocytochemical staining with an anti-vimentin antibody (Progen Biotechnik; GP53). Isolated Sertoli cells were cultured with F12-DMEM (Invitrogen, 10565–018) mixed with 10 μg/ml insulin (Nacalai Tesque, 19251–24), 5 μg/ml transferrin (Sigma, T1147), and 5 ng/ml epidermal growth factor (BD Bioscience, 40010) at 34°C. The culture medium was changed at days 2 and 4, and Sertoli cells were stimulated with 1 μM RA (Sigma) at day 5 for 24 hours. Then, qPCR analyses were performed.

### Protein extraction and western blotting

Mouse testes were homogenized in lysis buffer (20 mM Tris-HCl pH 8, 150 mM NaCl, 0.5 mM EDTA, 1% Triton X-100) containing a 1% protease inhibitor cocktail. The homogenates were centrifuged at 15,000 RPM for 5 min at 4°C, and the supernatant was separated by 10% SDS-PAGE and electroblotted onto PVDF membranes (GE Healthcare) under semi-dry conditions (Atto, Tokyo, Japan) as previously described [28]. The membranes were blocked with 5% skim milk and 1% bovine serum albumin (BSA) in PBST overnight at 4°C and then incubated with anti-MAFB antibody (1:1000; BETHYL IHC-00351) in blocking buffer for an hour at room temperature. After being washed, the membranes were then incubated with peroxidase-conjugated anti-rabbit IgG secondary antibody (Sigma, 1:2000) in blocking buffer for an hour at room temperature. As a loading control, we used an HRP-conjugated anti-β-actin antibody (1:3000; MBL PM057-7). Signals were detected via western blotting with luminol reagents (Nacalai). The protein marker used was WIDE-VIEW prestained protein size marker III (Wako, Osaka, Japan).

### Testosterone measurement

Male mice of each genotype (*n* = 4) were analyzed three times at 3 and 8 months of age. Blood was collected by cardiac puncture post-euthanasia, allowed to clot for 30 min, and centrifuged at 6,000 rpm for 10 min at 4°C to separate the plasma. Plasma testosterone concentration was determined using a testosterone ELISA kit from Enzo Life Sciences (Sapphire Bioscience; ADI-900-065) according to the manufacturer’s recommendations.

### Breeding test

Four adult males, 3 and 8 months old, of each genotype were mated with WT females (male/1 female). Females were subsequently observed for parturition 3 weeks (the gestation period of mice) after exposure to the male for a duration of 2 weeks. After the female mice gave birth, the number of pups per litter was recorded.

### Sperm analysis

Four adult male mice, 3 and 8 months old, of each genotype were evaluated. Total sperm obtained from the cauda epididymis were counted using a haemocytometer. Sperm motility parameters in samples containing >300 sperm were evaluated by loading the sample onto a microslide (0.1 × 2.0 mm; HTR 1099; VitroCom) and were measured using a TOX IVOS automated system (Hamilton Thorne).

### Proportion of seminiferous stages

Three male mice from each genotype were scarified; testes were dissected, fixed in 4%PFA, embedded in paraffin, and sectioned. The sections were then stained using periodic acid–Schiff (PAS) stain. In total, 100 seminiferous tubules were scored per animal. The tubules were staged as previously described [29].

### Flow cytometry

For *Mafb* expression analysis, adult *Mafb*-GFP knock-in mice in which the GFP gene was inserted into one allele of the *Mafb* locus [20] were used for the flow cytometric analysis as previously described [30]. Briefly, testes were treated twice with 1 mg/ml collagenase in DMEM containing 25 U/ml DNaseI twice at room temperature for 10 min and then with 2 mg/ml hyaluronidase and 1 mg/ml collagenase in DMEM containing 25 U/ml DNaseI at 32°C for 30 min with agitation. After filtration using a 35-μm filter, cells were suspended in PBS containing 2% fetal bovine serum (FBS) and 100 μg/ml propidium iodide (PI). PI-negative cells were sorted against GFP with a Beckman Coulter Gallios (Beckman Coulter), and Sertoli cells were distinguished from germ cells according to the parameters explained previously [30]. WT mice were used as a control. The data were analyzed using FlowJo software (Tree Star, Inc).

For RNA-Seq analysis, Sertoli cells from *Mafb*-cKO mice were sorted by the same method. *Mafb*^fl/fl^ mice were used as a control. The purity of the isolated cells was confirmed by immunocytochemical staining with anti-vimentin antibody (Progen Biotechnik; GP53).

### RNA sequencing and bioinformatic analysis

Total RNA was extracted using a Nucleospin RNA XS Kit (TaKaRa; 740902) from the isolated Sertoli cells of cKO and control mice at three months of age (*n* = 3, each group). The concentration and purity of the RNA were determined by automated optical density evaluation (OD 260/OD 280 ≥ 1.8 and OD 260/OD 230 ≥ 1.8) using a Nanodrop system. RNA sequencing libraries were prepared with a NEBNext rRNA Depletion Kit and an ENBNext Ultra Directional RNA Library Prep Kit (New England Biolabs) according to the manufacturer’s instructions using 9 to 70 ng of total RNA samples. Then, 2x36 base paired-end sequencing was performed with a NextSeq500 sequencer (Illumina) by Tsukuba i-Laboratory LLP (Tsukuba, Japan). FASTQ files were imported to the CLC Genomics Workbench (Version 7.5.1), mapped to the mm10 mouse genome and quantified for annotated genes in the ENSEMBLE database. FASTQ files containing the unmapped reads were deposited into NCBI GEO (accession no. GSE94297). Up-regulation and down-regulation were defined as relative transcription levels above Log2-fold change (FC) ≥ |±5|. Genes that were up-regulated or down-regulated by more than two folds were selected and filtrated by scatter plot. A heatmap was generated by R 3.2.1 GUI 1.66 Snow Leopard build (6956) software. The Database for Annotation, Visualization and Integrated Discovery (DAVID) was used for KEGG pathway analyses of the identified differentially expressed genes (DEGs) with thresholds of *P*-value <0.05 and enrichment gene count ≥3.

### Statistical analysis

Values were recorded as the mean±standard error (SEM) or ±standard deviation (S.D.) of the mean. The statistical significance of the differences among the means of several groups was determined using Student’s *t*-tests. *P*<0.05 was considered significant.

## Results

### MAF family of transcription factors in mouse testes

**Fig 1:**
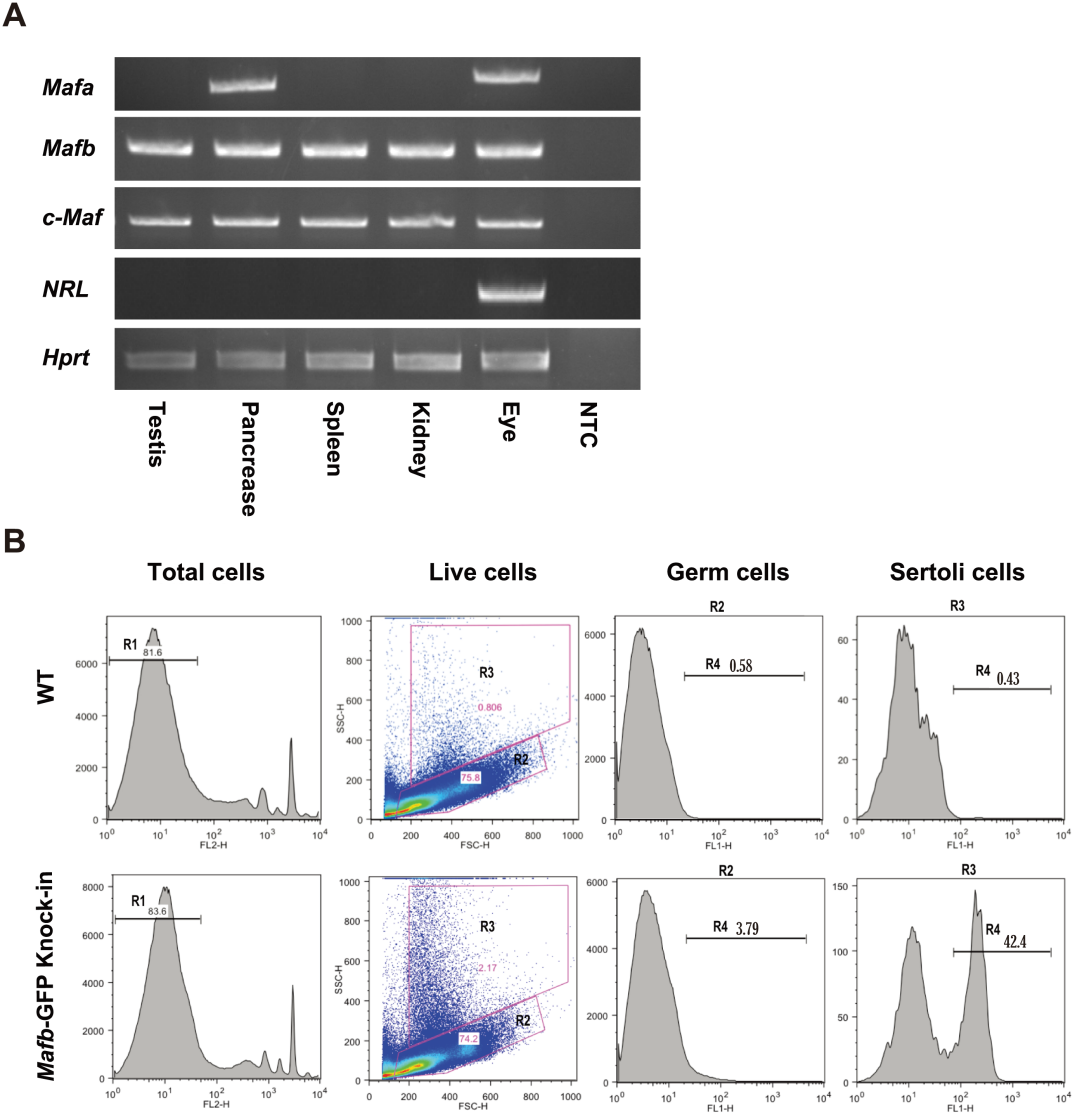
Expression of MAFB in mouse testes. (A) Reverse transcription-PCR (RT-PCR) of the large MAF transcription factors *Mafa*, *Mafb*, *c-Maf* and *Nrl* in various mouse tissues. Total RNA isolated from mouse testis, pancreas, spleen, kidney, and eye was used for RT-PCR analysis as described in the Materials and Methods section. RT-PCR of *Hprt* was carried out as a positive control. NTC was a non-template control. The RT-PCR analysis shown is representative of two independent experiments. (B) Detection of MAFB expression at Sertoli and germ cells in adult mouse testes. Testicular cells obtained from WT (upper panel) or *Mafb*-GFP knock-in mice (lower panel) were analyzed by FACS. Propidium iodide (PI)-negative cells (R1) were selected for flow sorting, while PI-positive dead cells were removed. The R1 fractions from WT or *Mafb*-GFP knock-in testicular cells were classified into two fractions, R2 representing germ cells and R3 representing Sertoli cells. GFP-positive cells are represented by the R4 fraction. The proportion of each fraction is shown above the bar.

The functions of genes are determined in part by their expression patterns. The expression of large MAF transcription factors in mouse testes has not been fully elucidated. To identify which MAF factor is expressed in testes, MAF mRNAs were analyzed by RT-PCR in adult mouse tissues. We extracted the total RNA from various mouse tissues, including the testes, pancreas, spleen, kidney, and eye. After synthesizing the cDNAs, PCR was performed for *Mafa*, *Mafb*, *c-Maf*, *Nrl*, and the internal control *Hprt*. The results showed that *Mafb* and *c-Maf* are the only large MAF transcription factors expressed in testes, in addition to their expression in the pancreas, spleen, kidney, and eye. *Mafa* was expressed in the pancreas and eye, while *Nrl* was only detectable in the eye (Fig 1A). Next, we focused on the transcription factor *Mafb*. To determine whether *Mafb* expression in testes was from somatic cells or germ cells within the seminiferous tubules, we used adult *Mafb*-GFP knock-in mice in which the GFP gene was inserted into one allele of the *Mafb* locus, and WT mice were used as the control. Testicular cells were analyzed by flow cytometry as explained previously in the Materials and Methods. Testicular live cells (R1 population) had two subpopulations: R2, which represented germ cells, and R3, which represented Sertoli cells (Fig 1B and Supplemental S1 Fig). The proportion of GFP-positive cells (R4) from both germ cells and Sertoli cells revealed that a small population (3.79%) of germ cells expressed *Mafb*, while 42.4% of the Sertoli cells expressed *Mafb* (Fig 1B).

**Fig 2:**
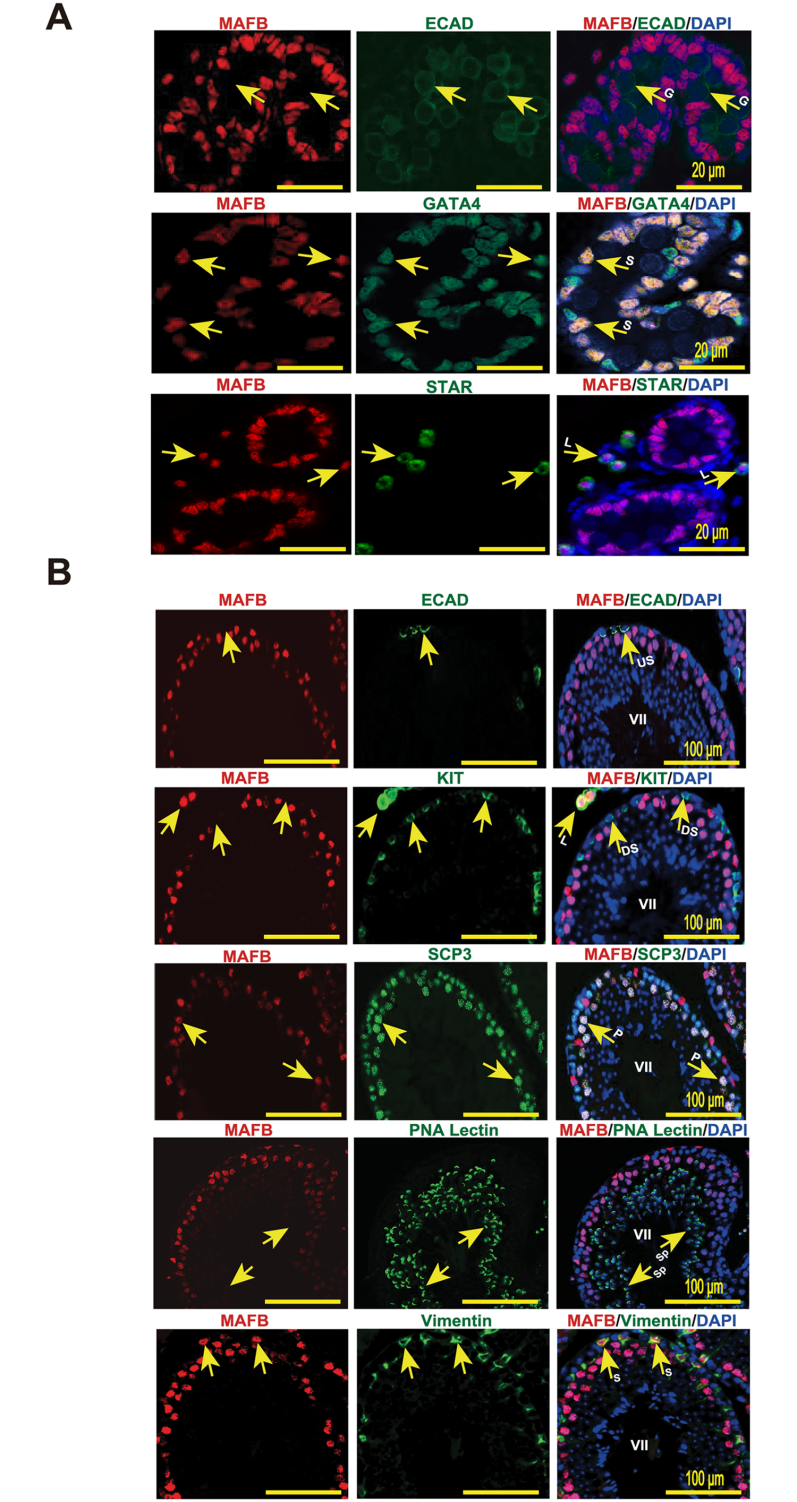
Localization of MAFB in mouse testes. (A) Localization of MAFB in E18.5 mouse testes. Double immunostaining of MAFB with E-cadherin (ECAD), GATA4, or STAR is shown. Nuclei were counterstained with DAPI. The color of each marker is indicated above the images. G; germ cells. S; Sertoli cells. L; Leydig cells. MAFB was specifically detected in Leydig cells and Sertoli cells. (B) Localization of MAFB in adult mouse testes. Double immunostaining of MAFB with E-cadherin (ECAD), KIT, SCP3, PNA Lectin, or vimentin is shown. Nuclei were counterstained with DAPI. The color of each marker is indicated above the images. All seminiferous tubules shown represent stage VII. US; undifferentiated spermatogonia. DS; differentiated spermatogonia. P; pachytene spermatocytes. Sp; spermatids. S; Sertoli cells. L; Leydig cells. MAFB was specifically detected in Leydig cells, Sertoli cells, and pachytene spermatocytes.

We then localized the expression of MAFB protein in mouse testis sections by immunohistochemistry using anti-MAFB antibody at the E18.5 and adult stage. At E18.5, MAFB protein was detected in Leydig cells, as indicated by MAFB co-localization with STAR^+^ cells outside seminiferous tubules, and in Sertoli cells, as indicated by MAFB co-localization with GATA4^+^ cells inside the tubules. In contrast, germ cells (E-Cadherin^+^) were negative for MAFB at the E18.5 stage (Fig 2A).

At the adult stage, Mafb was localized by staining of anti-MAFB together with markers for undifferentiated spermatogonia (E-cadherin), differentiated spermatogonia (KIT^+^ inside the tubule), spermatocytes (SCP3), spermatids (PNA-Lectin), Sertoli cells (vimentin), and Leydig cells (KIT^+^ outside the tubule) (Fig 2B). The results revealed that MAFB was localized at Leydig cells and Sertoli cells as indicated by MAFB co-localization with KIT^+^ cells outside the tubule and with vimentin, respectively. In addition, pachytene spermatocytes were found to be the only germ cells that expressed MAFB, as determined by MAFB co-localization with SCP3 cells and the morphology of their DNA. However, neither spermatogonia (ECAD^+^ or KIT^+^) nor other advanced germ cells, including spermatids (PNA-Lectin^+^) and testicular sperm expressed MAFB protein.

### Testis morphogenesis developed normally in *Mafb* KO mice

**Fig 3:**
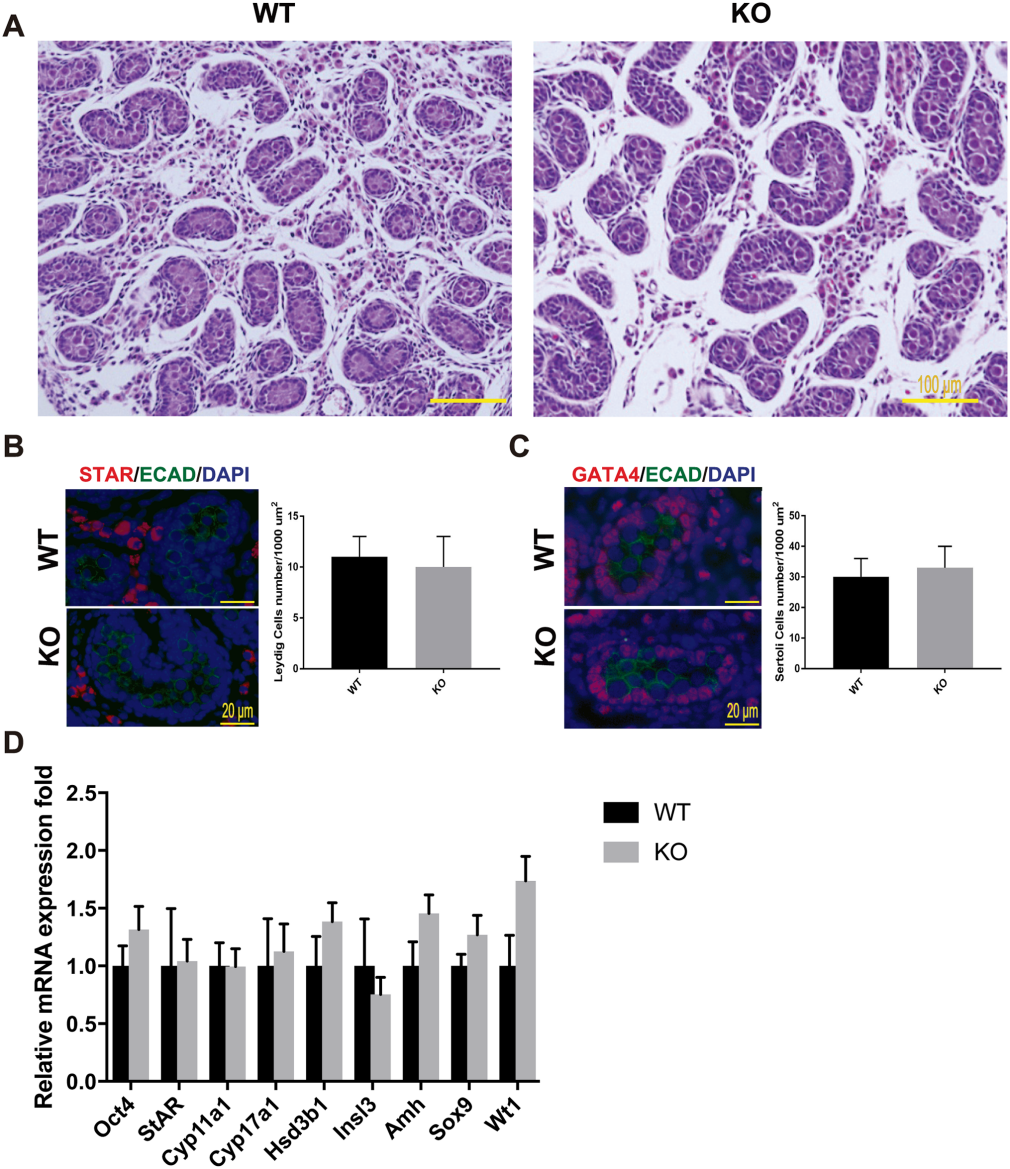
Testis morphogenesis of *Mafb* KO embryos developed normally. (A) Histological section of WT and KO E18.5 testes stained with HE. No morphological alteration or distribution was detectable. (B and C) Counts of Leydig and Sertoli cells from E18.5 WT and KO testes. Embryonic testes (*n* = 3 per genotype) were sectioned, and 4 sections for each gonad were randomly selected and stained with the germ cell marker E-cadherin (green) together with either STAR (red) or GATA4 (red). Numbers of STAR-positive cells outside seminiferous tubules (Leydig cells) or GATA4-positive cells inside the tubules (Sertoli cells) were counted per unit area. The values are the mean±S.D. *P<0.*05*. There was no significant difference between WT and KO cell counts. (D) The expression of genes involved in testes development and function. mRNA expression of the gene markers encoding for PGCs (*Oct4*), Leydig cells (*Cyp17a1*, *StAR*, *Insl3*, *Hsd3b1*, and *Cyp11a1*), and Sertoli cells (*Amh*, *Sox9*, and *WT1*) was determined by qRT-PCR in E18.5 WT and *Mafb* KO testes. Gene expression levels were normalized to *Hprt*. The bars represent the mean±SEM of five individuals. *P<0.05.

As described above, *Mafb* is expressed in embryonic testes. The effects of *Mafb* disruption on fetal testis morphogenesis were examined at the histological level and molecular level. We first analyzed the histology of prenatal mouse testes just before birth at E18.5 by staining with HE staining. The results showed that in for the *Mafb* KO mice, the seminiferous tubules were fully formed, and no abnormal structure was found in the testis sections (Fig 3A). Furthermore, Leydig and Sertoli cell numbers, the two types of cells that express *Mafb* in embryonic testes, were counted and shown as the number of cells per unit area (Fig 3B and 3C). Immunostaining for STAR (a Leydig marker) and GATA4 (a Sertoli marker within seminiferous tubules) were used as indicators. The average number of Leydig cells was 11±2 for WT *vs*. 10±3 in KO mice per 10000 μm^2^ area. The average number of Sertoli cells was 30±6 for WT *vs*. 33±7 in KO mice per 10000 μm^2^ area. In total, Leydig and Sertoli cell numbers did not differ significantly between KO and age-matched WT testes. In addition, no abnormal distribution or clustering of the Leydig cells was observed in the KO testes. To confirm and extend the histological data, the expression levels of cell type-specific genes involved in normal development and function of the testes were examined at E18.5 using qRT-PCR (Fig 3D). The expression level of *Oct4* (also known as *Pou5f1*), which is expressed exclusively in embryonic gonads by early pluripotent germ cells, was not altered between WT and KO testes. On the other hand, the expression levels of genes involved in steroid hormone synthesis as indictors of fetal Leydig cell development and function were examined in the KO testes. The steroidogenic protein levels of *StAR*, which facilitates the rapid transport of cholesterol into mitochondria, *Cyp11a1*, which catalyzes the conversion of cholesterol to pregnenolone, *Cyp17a1*, which catalyzes the conversion of pregnenolone into hydroxyprogesterone, and *Hsd3b1*, which converts pregnenolone to progesterone did not differ significantly between WT and KO testes, indicating that steroidogenic output was not compromised in the KO testes. Moreover, the expression of *Insl3*, which is produced by Leydig cells and is important for fetal testis descent, was unaltered. Similarly, the expression levels of the Sertoli cell markers *Amh*, *Sox9*, and *Wt1* were statistically unchanged between KO and WT mice. These data indicated that the development of the germ cell and somatic cell lineages in fetal testis are not disrupted in the absence of *Mafb*.

### MAFB in Sertoli cells is a stage-specific and induced by RA

**Fig 4:**
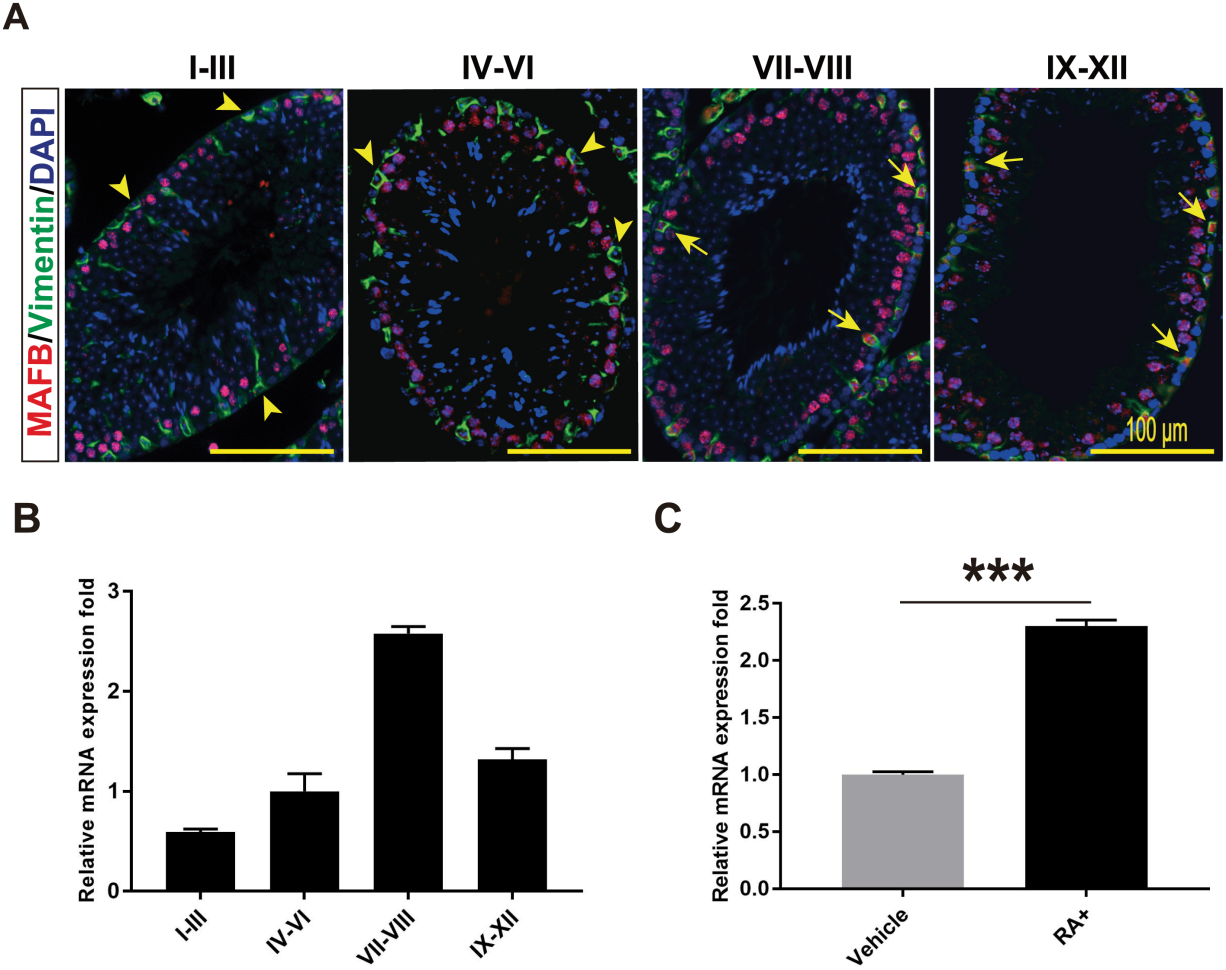
MAFB expression in seminiferous is stage specific and induced by RA. (A) Seminiferous tubules at stages I-III, IV-VI, VII-VIII and IX-XII were double immunostained with MAFB (red) and vimentin (green). Nuclei were counterstained with DAPI (blue). Arrowhead indicates MAFB-negative Sertoli cells, while Arrows indicated MAFB-positive Sertoli cells. MAFB in Sertoli cells was specifically detected in stages VII to XII. **(B)** Seminiferous tubules at stages I-III, IV-VI, VII-VIII and IX-XII were isolated from mature mouse testis, mRNAs were extracted, and the expression levels of *Mafb* were then compared by qRT-PCR. The proportion of stage-dependent *Mafb* expression is shown as columns. *Hprt* was used as an internal control. Bars represent the mean±S.D. (C) Cultured primary Sertoli cells were treated with 1 μM RA for 24 hours and expression changes in *Mafb* were quantified by qRT-PCR (*n* = 3). *Hprt* was used as an internal control. The bars represent the mean±S.D. ***P<0.001.

According to our FACS analysis explained above, not all Sertoli cells express MAFB. Therefore, to identify the reason for that heterogeneous phenomenon, we performed co-immunostaining of Sertoli cells marker vimentin together with MAFB for the different seminiferous stages: I-III, IV-VI, VII-VIII and IX-XII. We found that the expression of MAFB in Sertoli cells was not observed in stages I-VI but was highly detectable in stages VII-VIII followed by stages IX-XII (Fig 4A). This observation was further confirmed by isolating the different seminiferous stages from mature WT mice testis followed by RNA extraction and qRT-PCR. Similarly, the mRNA level of *Mafb* was dramatically increased in stages VII-VIII (2.58±0.07-fold) followed by stages IX-XII (1.32±0.11-fold) (Fig 4B). *Mafb* in stages I-III and IV-VI was 0.59±0.03-fold and 1.00±0.18-fold, respectively. These data indicate that *Mafb* expression in Sertoli cells is stage specific.

Next, because it is that stages VII-VIII are the stages in which A aligned spermatogonia transform into A1 under the control of RA [31–36], we examined whether *Mafb* is induced by RA synthesized in Sertoli cells. For this purpose, primary Sertoli cells were isolated and cultured in serum-free medium for 4 days followed by stimulation with RA at day 5. The expression changes in *Mafb* were quantified by qRT-PCR. The data revealed that the *Mafb* expression level was increased significantly by RA compared to that in control cells (Fig 4C). Altogether, *Mafb* showed the highest expression during stages VII-VIII, and the expression was increased by RA treatment in cultured Sertoli cells.

### Generation of *Mafb* conditional KO mice

We generated a tamoxifen-induced time-dependent deletion of conditional *Mafb* alleles. First, mice carrying floxed alleles of *Mafb* (*Mafb*^fl/fl^) were generated and then crossed with mice expressing Cre-ER^™^ recombinase (CAG-CreER^™^), which was driven by the chicken β-actin promoter and ubiquitously expresses the inducible Cre recombinase gene in all cell types [37].

**Fig 5:**
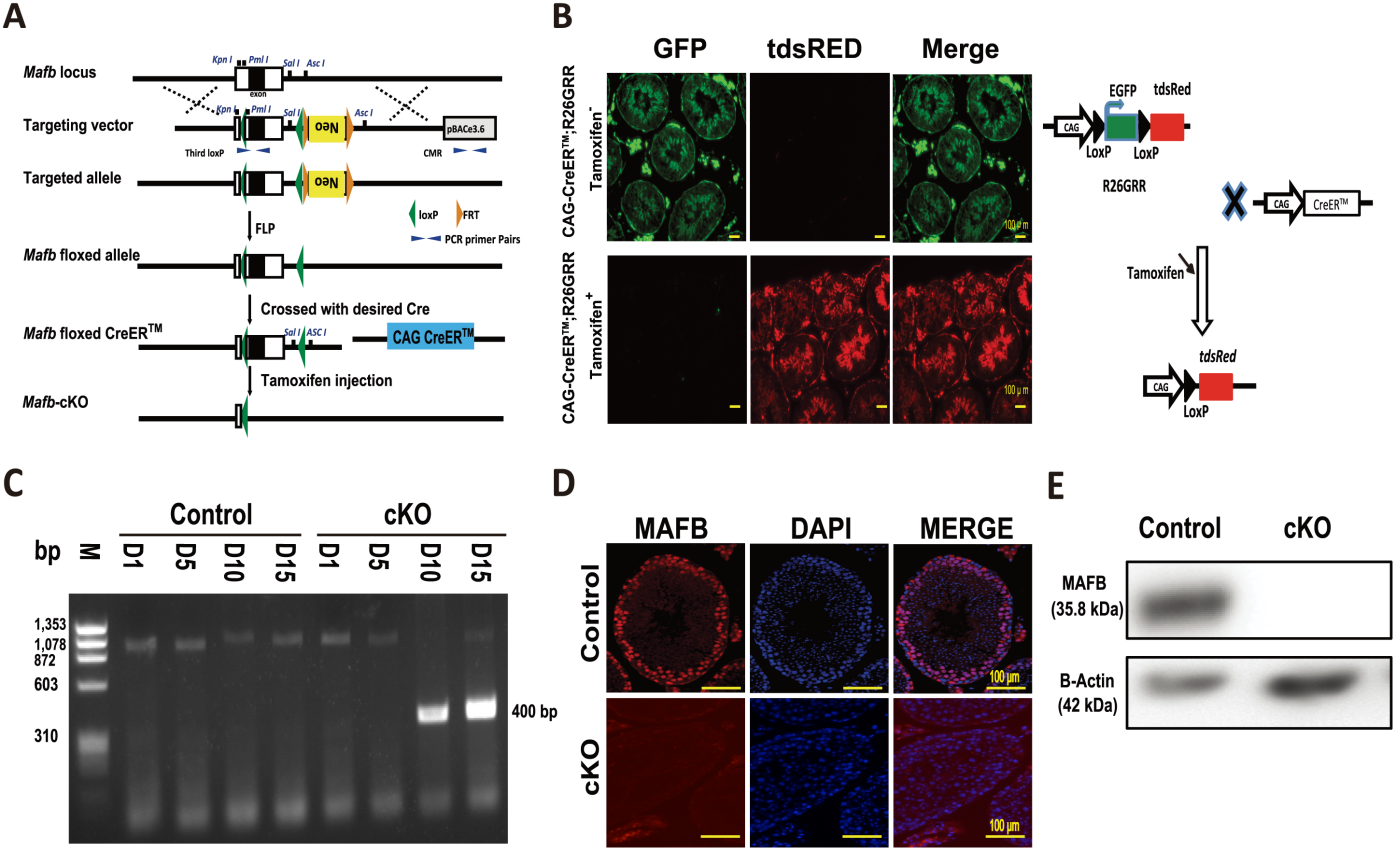
Generation of *Mafb*-cKO and efficiency evaluation. (A) Targeting strategy to generate *Mafb*^fl/fl^ CAG CreER^™^ (*Mafb*-cKO) mouse. The genomic structure of the mouse *Mafb* gene (top line) and illustrations of the targeting vector (second line) are shown. The *Mafb* single exon flanked by loxP sites (green triangles), and a neomycin (Neo) resistance cassette was inserted downstream flanked by FRT sites (orange triangles). The resultant targeted allele is shown (third line). The neomycin cassette was excised *in vivo* by crossing with the general FLP deleter strain, and the resultant floxed allele (fourth line) is shown. The floxed allele was crossed with the desired CAG-CreER^™^ (fifth line). The deleted allele after induction by tamoxifen is presented (bottom line). Restriction enzyme sites are shown in blue. PCR primers sites are indicated (blue triangles). (B) Efficiency of the Cre driver mice. The R26GRR reporter mouse, containing two cassettes EGFP flanked by loxP sites and tdsRED, was mated with the CAG-CreER^™^ driver mouse strain that ubiquitously expresses CreER^™^ in all cell types. The testes from a resultant male CAG-CreER^™^;R26GRR mouse were sectioned and analyzed pre- (upper panel) and post-tamoxifen injection (lower panel). The ability of the reporter mice to express tdsRED exclusively in testes was confirmed by microscopic examination of unstained sections. The mice crossing strategy is shown on the right. (C) Time-course of *Mafb* excision in the cKO mice. Six-week-old controls as well as cKO mice were intraperitoneally injected with tamoxifen. gDNA was extracted from mouse tails at post-tamoxifen injection days 1, 5, 10 and 15, and PCR was performed using a *Mafb*^*Δ*^ primer: 400 bp represents the deleted *Mafb* exon. D; Day. M; Marker. bp; base pair. (D) IHC was performed to verify MAFB deletion from cKO mouse testes after tamoxifen injection. Immunostaining for MAFB (red); nuclei were counterstained with DAPI (blue) as shown. The upper panel shows the control, while the lower panel shows cKO mouse testes. (E) Western blot confirming MAFB deletion in cKO mouse testes after tamoxifen injection. Total testis proteins were isolated, ~80 μg was loaded per well, and then the blot was probed with anti-MAFB or anti-β-actin antibody (internal control) for both the control and cKO mice, as shown. kDa; kilodaltons.

After two generations, we obtained mice carrying both *Mafb* floxed alleles and one copy of the Cre-ER^™^ transgene (*Mafb*^fl/fl^;CAG-CreER^™^). Mice of the *Mafb*^fl/fl^;CAG-CreER^™^ genotype are herein designated *Mafb*-cKO, and age-matched *Mafb*^fl/fl^ mice were considered controls (Fig 5A). To determine the efficiency of the Cre deleter line used, we crossed CAG-CreER^™^ driver mice with double-fluorescent Cre reporter mice R26GRR (*ROSA26*^*CAG-GFP/tdsRed*^) that express GFP fluorescence constitutively in all cell types prior to Cre-mediated excision and tdsRED after excision [21, 22]. The resulting male offspring were genotyped, and double heterozygous mice carrying GRR and Cre recombinase were then injected with tamoxifen at 6 weeks age. Two weeks later, the expression of green or red fluorescence in the testes was analyzed under a fluorescence microscope. Sections of the testes switched from green to red fluorescence, indicating that Cre-ER^™^ was efficient (Fig 5B). Next, to check the ablation of *Mafb* in the cKO mice, six-week-old mice were intraperitoneally injected with tamoxifen, and time-courses of Mafb excision from tail DNA were examined at post tamoxifen injection days 1, 5, 10 and 15 using *Mafb* excision primer (*Mafb*^*Δ*^). *Mafb* was found to be excised efficiently in adult mice starting from day 10 post-tamoxifen injection (Fig 5C). To further confirm MAFB deletion in the testes, two weeks following tamoxifen injection, mice were sacrificed, and MAFB expression in the testes was analyzed by immunohistochemistry (IHC) and western blotting. IHC results showed that no fluorescence signals were detected in cKO testes compared with those in the controls (Fig 5D). In addition, western blotting confirmed the absence of MAFB protein in the cKO mice testes (Fig 5E). These results indicated that *Mafb* conditional alleles were successfully generated and tamoxifen induces MAFB deletion in cKO mice.

### MAFB is dispensable for spermatogenesis maintenance in adult mouse testes

**Fig 6:**
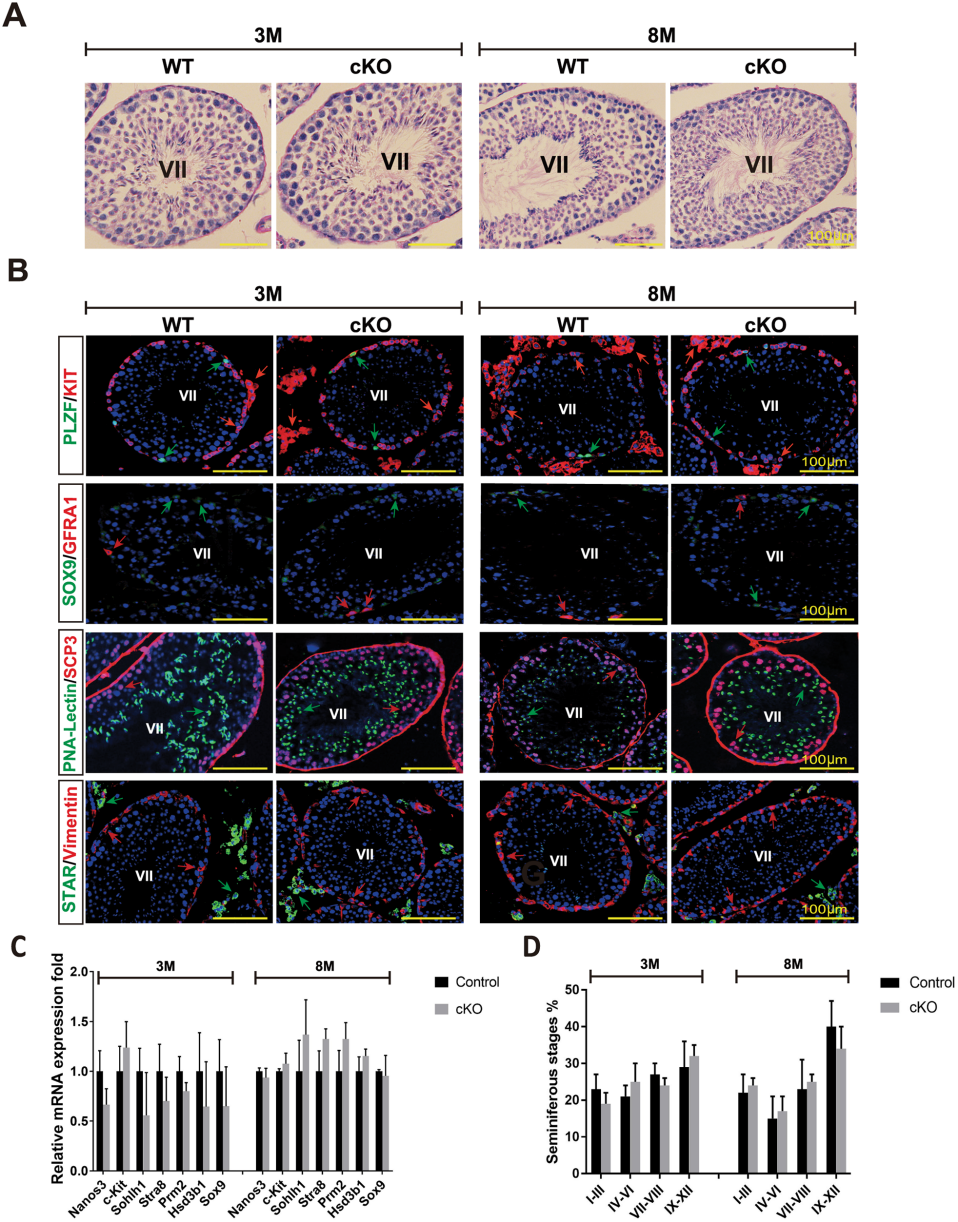
Spermatogenesis development of adult *Mafb*-cKO mice. Six-week-old mice were injected with tamoxifen and testes of cKO mice at 3- and 8-months of age were examined compared to those of age-matched controls (*n* = 4 for each group). (A) Testicular sections stained with Periodic acid-Schiff (PAS). (B) Immunostaining with various testicular cell types; undifferentiated spermatogonia (GFRA1 and PLZF), differentiated spermatogonia (KIT^+^ inside the tubule), spermatocytes (SCP3), spermatids (PNA-Lectin), Sertoli cells (SOX9 and Vimentin), and Leydig cells (KIT^+^ outside the tubule and STAR). The color of markers is indicated in the left boxes in panels. Colored arrows corresponding to the boxed markers are shown. (C) The expression level of the genes involved in testis function and germ cell development. The marker genes representing undifferentiated spermatogonia (*Nanos3*), differentiated spermatogonia (*c-Kit*), undifferentiated and differentiated spermatogonia (*Sohlh1*), differentiated spermatogonia and preleptotene spermatocytes (*Stra8*), spermatids (*Prm2*), Leydig cells (*Hsd3b1*), and Sertoli cells (*Sox9*) were analyzed by qRT-PCR. Each reaction was performed in duplicate for each gene. The data represent the means±SEM and are shown as relative mRNA expression after normalization to *Hprt*. *P<0.05. (D) The proportion of seminiferous stages: I-III, IV-VI, VII-VIII, and IX-XII from each genotype. *P<0.05. M; Month.

At first, we aimed to investigate whether MAFB is required for initiation of spermatogenesis in neonatal mice. For this purpose, we induced MAFB deletion before spermatogonia differentiation begins by tamoxifen injection on postnatal day 1 to 3. Unexpectedly, cKO mice died soon after MAFB deletion at postnatal days 5–10 (*unpublished data*). In contrast, at postnatal day 45 (adult stage), all mice were alive upon MAFB deletion, indicating that MAFB deletion led to neonatal death. Therefore, we analyzed whether MAFB deficiency would result in a disruption of spermatogenesis of adult mice. For this purpose, cKO and control mice (*n* = 4, per group) were injected with tamoxifen at 6 weeks of age and analyzed at 3 and 8 months of age. Each single animal was sacrificed, testes were dissected and histological sections were prepared and analyzed by PAS staining. The results showed that the seminiferous tubules had no abnormal morphological structure and exhibited complete spermatogenesis at both time points examined (Fig 6A). We then examined the absence of any testicular cell types by immunostaining with various cell type-specific markers. The results showed that the undifferentiated spermatogonia (GFRA1^+^ or PLZF^+^), differentiated spermatogonia (KIT^+^ inside the tubule), spermatocytes (SCP3^+^), spermatids (PNA-Lectin^+^), Sertoli cells (SOX9^+^ or vimentin^+^), and Leydig cells (KIT^+^ outside the tubule or STAR) were all present in the testicular sections from 3 and 8-month-old mice (Fig 6B). To confirm the histological data, the expression levels of cell type-specific genes involved in spermatogenesis development and testis functions were examined in 3- and 8-month-old cKO mice compared with age-matched control mice using qRT-PCR (Fig 6C). The expression levels of the following germ cell marker genes were not significantly different between cKO and control mice: *Nanos3*, expressed by undifferentiated spermatogonia; *c-Kit*, expressed by differentiated spermatogonia; *Sohlh1*, expressed by undifferentiated and differentiated spermatogonia; *Stra8*, expressed by differentiated spermatogonia and preleptotene spermatocytes with the onset of mitotic/meiotic switching; and *Prm2*, expressed at post-meiotic by haploid spermatids. Similarly, the expression level of the Leydig cell protein *Hsd3b1* involved in steroidogenic synthesis and the Sertoli cell protein *Sox9* were statistically unchanged between cKO and control mice. Furthermore, we examined whether there were any differences in the proportion of seminiferous stages in 3- and 8-month-old cKO mice compared with to age-matched controls (Fig 6D). Testis sections were stained by PAS staining and scored as explained in Materials and Methods. The data showed that the percentage of seminiferous stages at the examined ages were not significantly different. Collectively, we concluded that testicular function and spermatogenesis of the *Mafb*-cKO mice were apparently normal.

**Fig 7:**
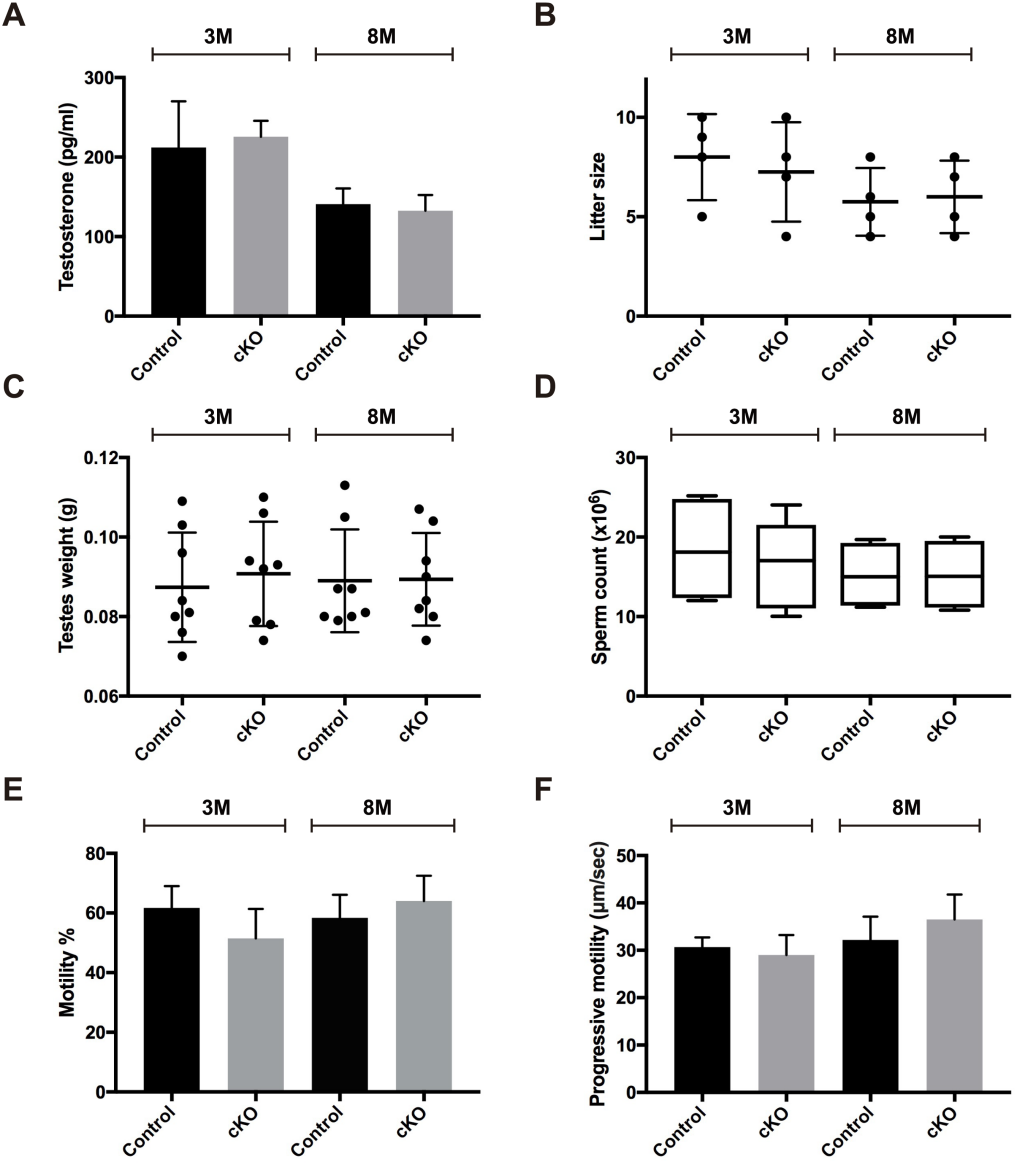
Examination of the testosterone levels and male fertility of adult cKO mice. Six-week-old cKO mice were injected with tamoxifen and examined at 3- and 8-months of age compared with age-matched controls (*n* = 4 for each group). **(A)** Blood plasma testosterone levels. (B) Litter size. (C) Testis weights. (D) Epididymal sperm counts. (E) Sperm motility %. (F) Progressive motility. M; Month. The data represent the mean±SEM. *P<0.05.

As MAFB is expressed in Leydig cells, we then examined if the lack of MAFB would affect the testosterone levels produced by Leydig cells, which is a mandatory hormone for spermatogenesis (Fig 7A). Therefore, we collected blood samples and checked plasma testosterone levels from 3- and 8-month-old male cKO *versus* age-matched control mice (*n* = 4, each group). There were no changes in plasma testosterone levels in either the 3-month-old mice (cKO = 225.56±20.07 *vs*. control = 212.23±57.83 pg/ml (mean±SEM); *P* = 0.8382) or the 8-month-old mice (cKO = 132.6±19.75 *vs*. control = 141±19.73 pg/ml (mean±SEM); *P* = 0.7802). Each single animal was then tested for fertility by mating with one WT C57BL/6J female. The cKO mice were shown to be fertile, with a litter size comparable to the age-matched control (Fig 7B). The mean pup number for the 3-month-old group was cKO = 7.25±1.25 *vs*. control = 8±1.8 pups (mean±SEM; *P* = 0.0.6658), and the 8-month-old group was cKO = 6±0.91 *vs*. control = 5.75±0.85 pups (mean±SEM; *P* = 0.8481). The mice from 3- and 8-month-old groups were then sacrificed, testis weight was measured, and the cauda epididymis was dissected for sperm analyses. The results showed that testis weight, sperm count, sperm motility, and progressive motility were not statistically different between cKO and age-matched control mice (Fig 7C–7F). Together these data illustrate that MAFB is not required for spermatogenesis maintenance in adult mice.

### Changes in the transcriptome profile of *Mafb*-cKO Sertoli cells

Since MAFB is a transcription factor, we were interested in examining whether the expression of the transcriptome of Sertoli cells was affected by the absence of MAFB. Sertoli cells were sorted from 3-month-old cKO as well as age-matched control mice (*n* = 3, each group). Total RNA was extracted from the isolated cells, and RNA samples (pooling for WT or cKO, *n* = 3 each) were analyzed by RNA-Seq. The purity of the isolated cells was confirmed by immunostaining using a Sertoli cell-specific marker (S2A Fig). The peaks representing RNA-Seq tag counts of the *Mafb* single exon confirmed the reduction of *Mafb* in cKO Sertoli cells (S2B Fig). RNA-Seq analysis showed that 1937 transcripts with a fold change ≥2 in cKO Sertoli cells were differentially regulated, and among them, 1103 were down-regulated and 834 were up-regulated. Detailed information regarding the down-regulated and up-regulated transcripts is listed in S1 and S2 Tables, respectively. *Gng11*, *Nck2*, *Prr3*, *Tlr1*, and *Nlrp3* were randomly selected and examined by qRT-PCR which validated the RNA-Seq data (S2C Fig).

DAVID Database was used for KEGG pathway analyses of the identified DEGs with fold change ≥2 in *Mafb*-cKO Sertoli cells as indicated in Table 2. The pathways with *P*-value <0.05 and enrichment gene count ≥3 of the down- and up-regulated genes were not related to reproduction. Notably, the identified down regulated genes were related to immunodeficiency and phagocytosis activity of Sertoli cells, as revealed in Table 2.

**Table 2:** KEGG pathway analysis of the DEGs in *Mafb*-cKO Sertoli cells.

KEGG_id	Description	Count	List Total	Genes	P-value
**Down-regulated**					
mmu04620	Toll-like receptor signaling pathway	21	303	*PIK3CG*, *CCL3*, *IL6*, *LY96*, *TLR1*, *CXCL9*, *TLR2*, *NFKBIA*, *FADD*, *CD40*, *TLR6*, *CCL4*, *TLR7*, *CXCL10*, *CD86*, *RIPK1*, *MAP3K8*, *PIK3R5*, *CD14*, *AKT3*, *SPP1*	1.40E-07*
mmu04060	Cytokine-cytokine receptor interaction	33	303	*CCL3*, *CCL2*, *TNFRSF25*, *TNFRSF12A*, *CSF2RB2*, *CCR1*, *IL18*, *IL21R*, *CXCL2*, *CCL9*, *CXCL9*, *PF4*, *CCL4*, *CCL7*, *IL10*, *CXCL10*, *CCL6*, *CCL24*, *TNFRSF1B*, *TNFRSF11A*, *IL10RA*, *CSF3R*, *PDGFC*, *IL13RA1*, *IL1A*, *IL6*, *BMP2*, *TNFRSF17*, *CD40*, *GM614*, *OSM*, *ACVR2B*, *CX3CR1*	1.10E-06*
mmu03010	Ribosome	18	303	*GM7290*, *GM7808*, *GM12174*, *GM3362*, *RPL36A*, *GM12034*, *GM5786*, *GM8730*, *GM5621*, *GM14173*, *GM6030*, *GM10036*, *GM4149*, *GM14323*, *RPL21*, *GM15772*, *GM14217*, *GM10132*, *RPS27A*, *GM15500*, *GM11942*, *GM10269*	2.80E-06*
mmu04062	Chemokine signaling pathway	25	303	*CCL3*, *CCL2*, *PREX1*, *CCR1*, *CXCL2*, *CCL9*, *CXCL9*, *NFKBIA*, *PF4*, *CCL4*, *CCL7*, *PRKX*, *CCL6*, *CXCL10*, *CCL24*, *PTK2*, *RAC2*, *PIK3R5*, *AKT3*, *PIK3CG*, *LYN*, *HCK*, *WAS*, *CX3CR1*, *JAK2*	2.40E-05*
mmu04210	Apoptosis	13	303	*PIK3CG*, *CFLAR*, *CSF2RB2*, *RIPK1*, *NTRK1*, *CASP12*, *EXOG*, *NFKBIA*, *FADD*, *PIK3R5*, *PRKX*, *IL1A*, *AKT3*	1.80E-03*
mmu05322	Systemic lupus erythematosus	14	303	*C3*, *GM14474*, *HIST1H2BG*, *HIST1H2BH*, *SNRPD1*, *FCGR4*, *SSB*, *CD40*, *IL10*, *TRIM21*, *C8G*, *CD86*, *FCGR2B*, *GM14484*	2.70E-03*
mmu04621	NOD-like receptor signaling pathway	10	303	*IL6*, *CARD9*, *CCL2*, *NAIP2*, *IL18*, *CXCL2*, *NFKBIA*, *NLRP3*, *CASP1*, *CCL7*	4.70E-03*
mmu04662	B cell receptor signaling pathway	11	303	*PIK3CG*, *CD19*, *LYN*, *RAC2*, *FCGR2B*, *NFKBIE*, *NFKBIA*, *PIK3R5*, *AKT3*, *BLNK*, *NFATC1*	8.70E-03*
mmu05340	Primary immunodeficiency	7	303	*GM614*, *DCLRE1C*, *PTPRC*, *CD19*, *CD4*, *CD40*, *BLNK*	1.00E-02*
mmu04370	VEGF signaling pathway	10	303	*PIK3CG*, *PTK2*, *PTGS2*, *RAC2*, *JMJD7*, *MAPKAPK3*, *PIK3R5*, *PLA2G2D*, *AKT3*, *NFATC1*	1.80E-02*
mmu00520	Amino sugar and nucleotide sugar metabolism	7	303	*RENBP*, *UAP1*, *HK3*, *GFPT2*, *PGM1*, *UGDH*, *UXS1*	2.60E-02*
mmu04666	Fc gamma R-mediated phagocytosis	11	303	*PIK3CG*, *PTPRC*, *PLD1*, *LYN*, *RAC2*, *FCGR2B*, *HCK*, *RPS6KB2*, *PIK3R5*, *WAS*, *AKT3*	3.20E-02*
mmu00500	Starch and sucrose metabolism	6	303	*GANC*, *UGT1A6B*, *HK3*, *PGM1*, *UGDH*, *UXS1*	3.90E-02*
mmu04610	Complement and coagulation cascades	9	303	*PLAT*, *C3AR1*, *C5AR1*, *C3*, *F13A1*, *SERPINE1*, *CFH*, *PLAUR*, *C8G*	4.20E-02*
mmu04660	T cell receptor signaling pathway	12	303	*PIK3CG*, *PTPRC*, *NFKBIE*, *NCK1*, *MAP3K8*, *NFKBIA*, *CD4*, *PIK3R5*, *IL10*, *AKT3*, *TEC*, *NFATC1*	4.50E-02*
**Up-regulated**					
mmu05016	Huntington's disease	12	178	*DNAH7B*, *NDUFB3*, *DNAH10*, *NDUFA5*, *NDUFB4*, *COX8C*, *CYCT*, *DNAH17*, *COX7C*, *DNAH1*, *DNAIC1*, *NDUFB2*	5.70E-03*
mmu01100	Metabolic pathways	42	178	*INPP1*, *NDUFB3*, *NDUFB4*, *SEC1*, *GBGT1*, *COX7C*, *AASS*, *TKTL2*, *GPAT2*, *NDUFB2*, *PLCH2*, *PEMT*, *FUT4*, *ENO3*, *B3GNT3*, *CSL*, *PCYT2*, *B4GALT7*, *ACSL6*, *DCTPP1*, *NDUFA5*, *COX8C*, *CYCT*, *MCAT*, *FBP1*, *DGKH*, *POLR1C*, *CDO1*, *LPCAT4*, *APRT*, *NME5*, *PLCG1*, *PTGDS*, *PYGM*, *RRM2*, *DPM2*, *MTAP*, *PLA2G2C*, *SMPD4*, *SMPD3*, *NMNAT1*, *DUT*	1.03E-02*
mmu03460	Fanconi anemia pathway	5	178	*SLX4*, *SLX1B*, *FANCG*, *UBE2T*, *ERCC1*	2.90E-02*
mmu03013	RNA transport	9	178	*AAAS*, *NUP62*, *RASL2-9*, *PABPC2*, *THOC6*, *THOC7*, *NUP188*, *NUPL2*, *POM121L2*	4.10E-02*

Count, number of the down-regulated genes involved in a pathway; KEGG, Kyoto Encyclopedia of Genes and Genomes.

* Statistically significant, *P*<0.05.

## Discussion

The present study was undertaken in an effort to define the physiological role of the *Mafb* gene in mammalian testes. MAFB is a transcription factor that plays an important role in regulating the development and differentiation of various tissues. A previous study of embryonic mouse gonads showed that MAFB is expressed in the XY gonads along the gonad-mesonephros border as early as E11.5 and is then restricted to Leydig cells by E14.5 [10]. Subsequent studies have used *Mafb*-GFP knock-in as an early marker of the Leydig cell lineage in mice [38–40]. Whether MAFB is necessary for embryonic testis morphogenesis or not is unknown. Just before birth at E18.5, we localized MAFB expression and found that MAFB is expressed in Sertoli cells in addition to being expressed in Leydig cells. However, examination of E18.5 *Mafb* KO embryos via somatic cell counts, histological analysis, and molecular analysis indicated that testis morphogenesis was normal. At the adult stage, it has been reported that the active metabolite of vitamin A (RA) is essential for initial differentiation and meiotic entry of mouse spermatogonia [11]. Experiments using vitamin A-deficient mice resulted in a blockade of A to A1 spermatogonia transition, and spermatogenesis could be recovered upon RA treatment [13]. More precisely, RA drives spermatogonia differentiation in two ways: the first spermatogenic wave due to RA from Sertoli cells and subsequent waves due to RA from spermatocytes [11, 14–19]. However, the downstream targets of RA are still unknown. As the evidence that the large MAF transcription factor in *Drosophila melanogaster*, *TJ*, which encodes an orthologue of the typical bZIP transcription factors MAFB and c-MAF in vertebrates, was shown to be mandatory for germ cell differentiation in Drosophila [9]. In addition, RA was reported to induce MAFB expression and there is a robust RAR-binding site at the end of the *Mafb* coding region [11, 41]. Therefore, MAFB might have a conserved function between species to induce spermatogonial differentiation under the control of RA. In this regard, we found that MAFB is localized in Sertoli cells and pachytene spermatocytes in postnatal mouse seminiferous tubules, as confirmed by immunohistochemical staining and flow cytometric analyses. The expression of MAFB protein in Sertoli cells was specific to stages VII-XII but was not observed in stages I-VI, which can be explained by the low mRNA expression level of *Mafb* at stages I-VI. Along the same line, it is well known that stages VII-VIII, in which *Mafb* was highly expressed, are the stages of A aligned spermatogonia transformation into A1 under the control of RA [31–36]. Our observations were consistent with the hypothesis that the transcription factor MAFB may take part in the regulation of the onset of the spermatogenic cycle.

To analyze this hypothesis, we generated a time-dependent deletion of *Mafb* alleles via tamoxifen treatment, as MAFB KO mice die by birth due to respiratory failure [42]. Surprisingly, our data revealed that adult cKO mice were fertile and did not exhibit any abnormality in testicular histology, proportion of seminiferous stages, expression levels of germ cell markers, or plasma testosterone levels, indicating that MAFB is dispensable for spermatogenesis. Similarly, GATA-1 expression in Sertoli cells, which is induced in response to the cyclical changes of seminiferous stages, specifically during stages VII-IX, was found to be dispensable for spermatogenesis as well [43, 44]. Many other examples of genes expressed in testes with dispensable function have been reported [44–54]. Several possible explanations may account for these phenomena. First, spermatogenesis is a complex process that involves many genes, and the inactivation of one gene may not be sufficient to induce a detectable phenotype [55]. Consistently, we found that both *Mafb* and *c-Maf* are expressed in postnatal testes and that c-MAF protein may compensate for the function of MAFB, as in macrophages [56, 57]. Thus, double knockout mice should be further evaluated. Second, the defects in *Mafb*-cKO mouse testes may be too small to be detected using the techniques employed here. A detailed ultrastructural examination of testicular tissues may be needed to confirm any minor changes in cKO mice. Third, the up-regulation of the *Mafb* expression started at stage VII is a dramatic response to the RA signaling that is periodically activated during stages VII-XII, and this activation alters the expression of various Sertoli cell's genes [13, 30].

Next, we aimed to investigate whether MAFB is required to establish the first spermatogenic wave in neonatal mice testis. Before the spermatogonia differentiation started, we induced MAFB deletion by tamoxifen injection on postnatal days 1 to 3. Spermatogonia are known to begin to differentiate between postnatal days 6–10 [58–60]. Unexpectedly, the cKO mice died shortly after MAFB deletion between postnatal days 5–10 and therefore could not be reliably analyzed. However, as Sertoli cells could not initiate the first spermatogenic wave in adult VAD mice until RA recovered, we hypothesized that if MAFB in Sertoli cells acts downstream of RA to initiate the first spermatogenic wave, the transcriptome profile of *Mafb*-cKO Sertoli cells would be affected. Thus, we analyzed the transcriptome profile of Sertoli cells by RNA sequencing. Signaling pathway analyses of the DEGs were not related to spermatogenesis. However, the down-regulated transcripts were related to immunodeficiency and phagocytosis pathways. These data could be the fourth explanation for the possible function of MAFB as a phagocytic modulator in Sertoli cells during the identified positive seminiferous stages. In this connection, it is known that during spermatogenesis half of differentiating germ cells undergo apoptosis before maturing into spermatozoa [61–63]. Since macrophages, the integral compartment of the immune system, are absent inside the seminiferous tubules, Sertoli cells that are endowed with the phagocytic properties respond to the inflammatory signals by rapid engulfment of the apoptotic germ cells within the seminiferous epithelium [63–67]. Further studies should clarify if MAFB, an important modulator of macrophage phagocytosis, is involved in such process in Sertoli cells [68–70]. Moreover, the possibility that MAFB might be required for initiation of the first spermatogenic wave in neonatal testis should be clarified using a Sertoli cell-specific Cre driver mouse.

## Conclusions

The transcription factors MAFB and c-MAF are expressed in mouse testes. MAFB localized in Leydig and Sertoli cells in testes at E18.5 while it localized in Leydig cells, Sertoli cells, and pachytene spermatocytes in adult testes. Our current examination technique revealed that MAFB-deficient testes developed normally by E18.5, and spermatogenesis was not disrupted in adult mice. The transcriptome profile of adult cKO Sertoli cells showed the down-regulation of genes related to the immune function and phagocytosis activity of Sertoli cells.

## Supporting information

S1 Table List of the down-regulated transcripts of *Mafb*-cKO Sertoli cells. (XLSX)

S2 Table List of the up-regulated transcripts of *Mafb*-cKO Sertoli cells. (XLSX)

S1 Fig Sertoli and germ cell separation by FACS. Immunostaining with the Sertoli cell marker vimentin (red) before and after cell sorting. Upper panels (R1): before sorting. Middle (R2) and Lower (R3) panels: after sorting. The R2 population represents germ cells as vimentin negative, while the R2 population represents Sertoli cells as vimentin positive. Nuclei were counterstained with DAPI (blue). The proportion of vimentin-positive cells is shown in the R3 merged photo. The value is the mean±S.D.
(TIF)

S2 Fig RNA-Seq validation for the transcriptome profile of *Mafb*-cKO Sertoli cells. Sertoli cells were sorted from three-month-old *Mafb*-cKO or control mice and analyzed by RNA-Seq. (A) The purity of the isolated cells was confirmed by IHC staining with the Sertoli cell-specific marker anti-vimentin antibody (red) and nuclei were counterstained with DAPI (blue). The proportion of vimentin-positive cells is shown in the merged photo. (B) UCSC genome browser screenshot showing the RNA-Seq tag counts. Reads from both genotypes were mapped to the single exon of the *Mafb* gene and visualized to confirm the reduction in the cKO reads. The full genome sequence for Mus musculus provided by UCSC (mm10, Dec. 2011) was used for mapping by HISAT. The arrow under the panel shows the exon and indicates the direction of transcription. Each panel is labeled with the genotype. The read histograms show the number of reads at each nucleotide. (C) qRT-PCR validation for the RNA-Seq data analyses. Expression of five differentially expressed genes (up and down) that were randomly selected are shown.
(TIF)
