# Supplementary figures and images for: MAFB is dispensable for the fetal testis morphogenesis and the maintenance of spermatogenesis in adult mice
Source: PLoS One. 2018 Jan 11;13(1):e0190800. doi: 10.1371/journal.pone.0190800 (PMC5764304; doi:10.1371/journal.pone.0190800)

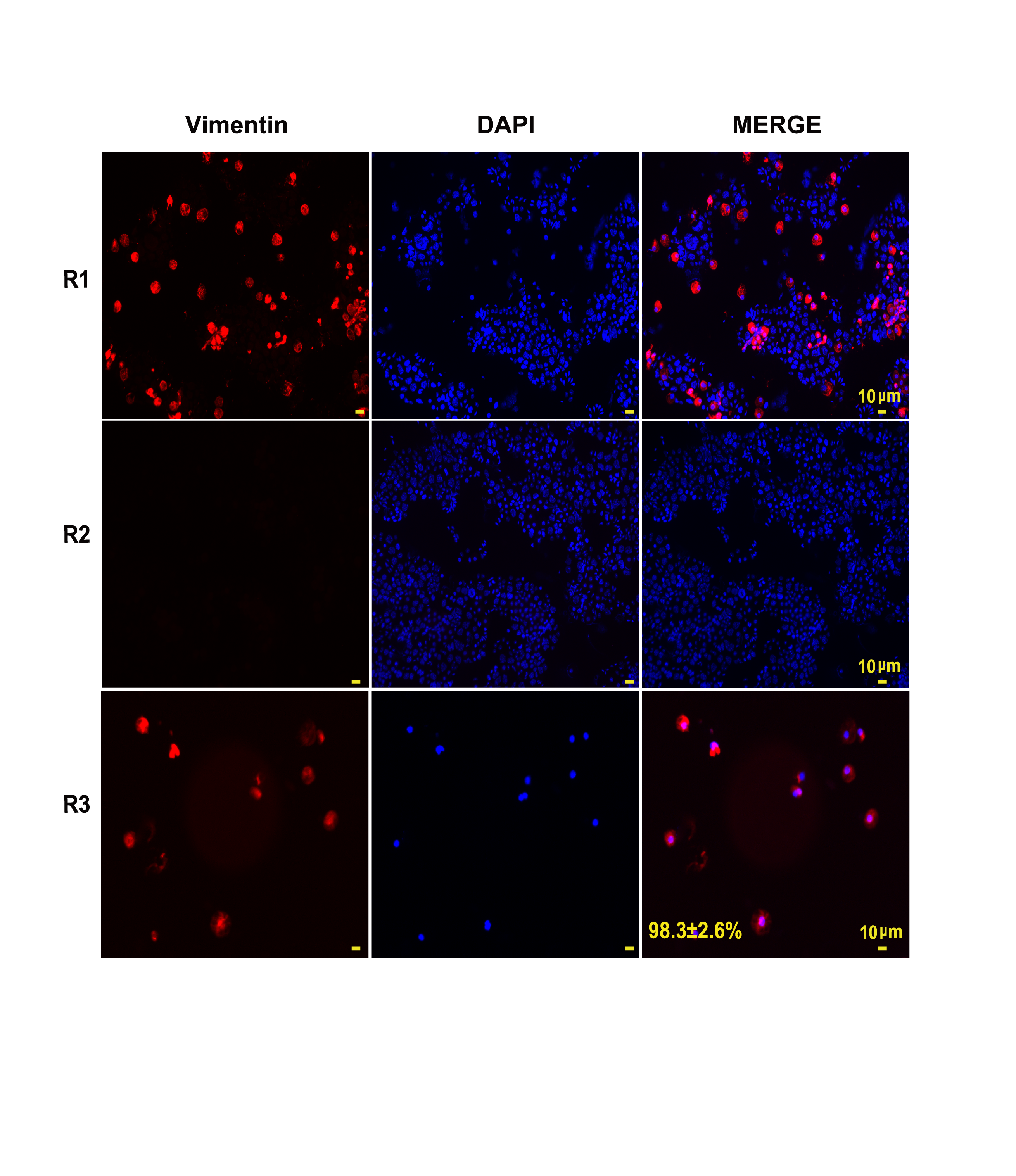

Supplement: S1 Fig — Immunostaining with the Sertoli cell marker vimentin (red) before and after cell sorting. Upper panels (R1): before sorting. Middle (R2) and Lower (R3) panels: after sorting. The R2 population represents germ cells as vimentin negative, while the R2 population represents Sertoli cells as vimentin positive. Nuclei were counterstained with DAPI (blue). The proportion of vimentin-positive cells is shown in the R3 merged photo. The value is the mean±S.D. (TIF) [file pone.0190800.s003.tif]

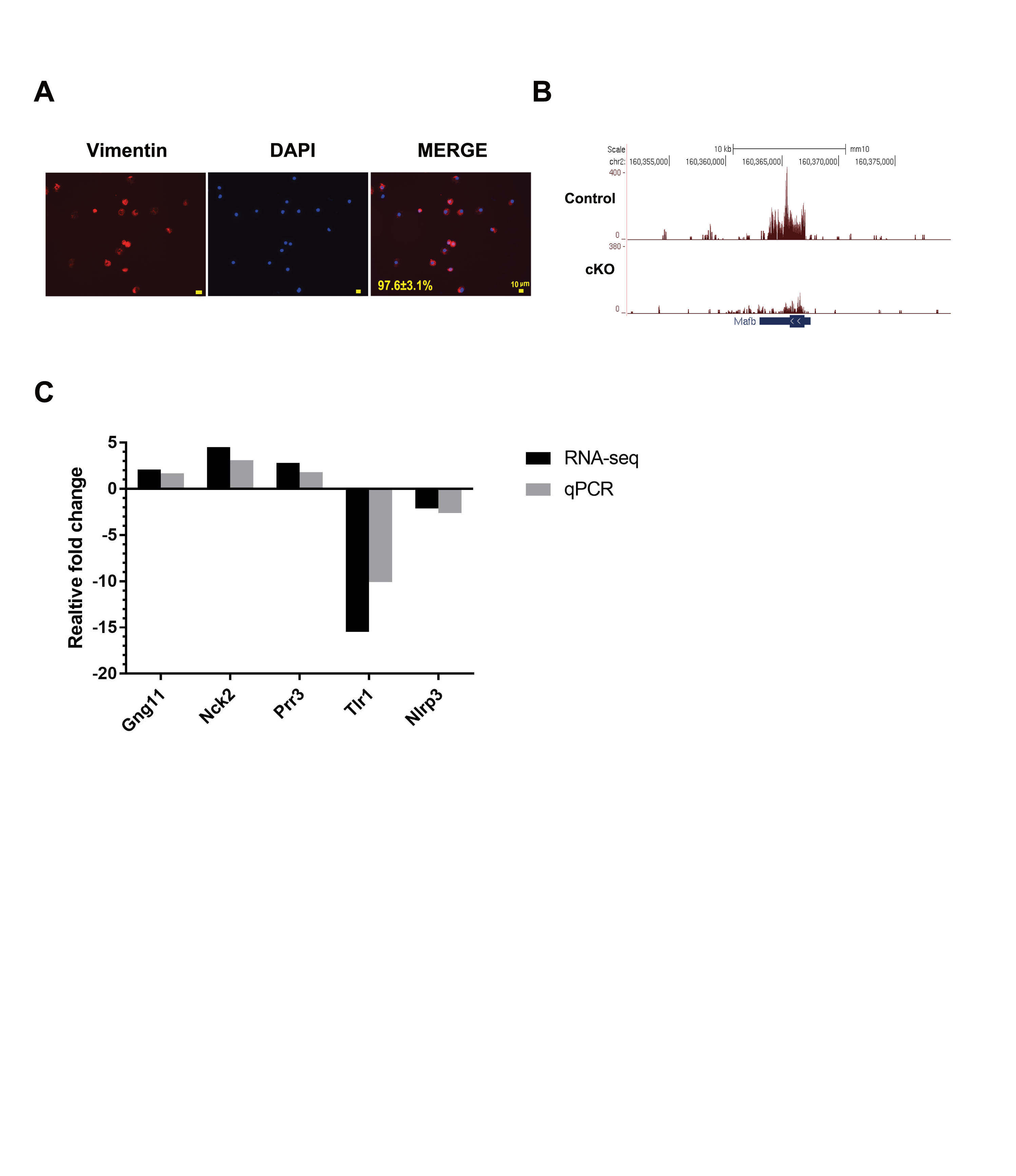

Supplement: S2 Fig — Sertoli cells were sorted from three-month-old Mafb-cKO or control mice and analyzed by RNA-Seq. (A) The purity of the isolated cells was confirmed by IHC staining with the Sertoli cell-specific marker anti-vimentin antibody (red) and nuclei were counterstained with DAPI (blue). The proportion of vimentin-positive cells is shown in the merged photo. (B) UCSC genome browser screenshot showing the RNA-Seq tag counts. Reads from both genotypes were mapped to the single exon of the Mafb gene and visualized to confirm the reduction in the cKO reads. The full genome sequence for Mus musculus provided by UCSC (mm10, Dec. 2011) was used for mapping by HISAT. The arrow under the panel shows the exon and indicates the direction of transcription. Each panel is labeled with the genotype. The read histograms show the number of reads at each nucleotide. (C) qRT-PCR validation for the RNA-Seq data analyses. Expression of five differentially expressed genes (up and down) that were randomly selected are shown. (TIF) [file pone.0190800.s004.tif]
